# A Retrospective Cross-Sectional Study on the Risk of Getting Sick with COVID-19, the Course of the Disease, and the Impact of the National Vaccination Program against SARS-CoV-2 on Vaccination among Health Professionals in Poland

**DOI:** 10.3390/ijerph19127231

**Published:** 2022-06-13

**Authors:** Sylwia Kałucka, Ewa Kusideł, Izabela Grzegorczyk-Karolak

**Affiliations:** 1 Department of Coordinated Care, Medical University of Lodz, 90-251 Lodz, Poland; 2 Department of Spatial Econometrics, Faculty of Economics and Sociology, University of Lodz, 90-255 Lodz, Poland; ewa.kusidel@eksoc.uni.lodz.pl; 3 Department of Biology and Pharmaceutical Botany, Medical University of Lodz, 90-151 Lodz, Poland; izabela.grzegorczyk@umed.lodz.pl

**Keywords:** awareness of the risk, disease COVID-19, SARS-CoV-2 vaccine, exposure, healthcare workers, health behavior, infectious diseases, SARS-CoV-2 National Vaccination Program, sources of information, vaccination decision

## Abstract

Six months after starting the National Vaccination Program against COVID-19, a cross-sectional retrospective study was conducted among 1200 salaried and non-salaried healthcare workers (HCWs) in Poland. Its aim was to assess factors including the risk of exposure to COVID-19, experiences with COVID-19, the trust in different sources of knowledge about the pandemic and SARS-CoV-2 vaccines, and the government campaign on vaccination as predictors of vaccination acceptance. The strongest awareness of a high risk of work-associated infection was demonstrated by doctors (D) (72.6%) and nurses and midwives (N) (64.8%); however, almost half of the medical students (MS) and nursing and midwifery students (NS) did not identify as a risk group. Out of several dozen variables related to sociodemographic characteristics and personal experience of COVID-19, only occupation, previous COVID-19 infection, and high stress seemed to significantly influence vaccination acceptance. Interestingly, only 6.7% of respondents admitted that the government campaign impacted their decision to vaccinate. This result is not surprising considering that the vast majority of respondents (87.8%) learned about vaccinations from sources such as academic lectures (29.9%), health professionals (29.0%), or the internet (28.9%). Those who gained information about vaccination from traditional media (radio, television, and daily press), a popular platform of the government campaign, had a lower propensity to vaccinate (OR = 0.16, *p* < 0.001). Additionally, almost twice as many considered the information provided in the campaign to be unreliable. Our findings, from this retrospective study, do not confirm that the government campaign was effective for healthcare professionals. Therefore, in this group, other forms of vaccination incentives should be sought. However, the vaccinated respondents were significantly more likely to support compulsory vaccination against COVID-19 among health professionals.

## 1. Introduction

The COVID-19 pandemic is a situation that the present generation has never met before. By May 2022 [1], the SARS-CoV-2 had infected over 525 million people in more than 190 countries and caused more than 6.285 million deaths globally. In order to manage the COVID-19 pandemic and avoid any potential recurrence, considerable interdisciplinary cooperation has been underway. Less than 1 year after the World Health Organization announced the COVID-19 global pandemic, in March 2020, many countries began mass SARS-CoV-2 virus vaccination programs [1]. In many cases, the first to be eligible for vaccination were healthcare workers (HCWs) and individuals at higher risk of COVID-19 complications and death. There is no doubt that HCWs constitute a priority group [2]; they experience continuous exposure to infected and asymptomatic patients in the workplace, as well as experience virus transmission from other HCWs. However, as estimated, HCWs demonstrated varying levels of acceptance for the SARS-CoV-2 vaccination before the vaccines were released, ranging from 27.7% acceptance in the Democratic Republic of the Congo to 86% in Italy or even more in South Africa [3,4,5]. Additionally, differences occurred among different professions, as physicians demonstrated a higher level of confidence in vaccines than nurses [6].

Due to the rapid spread of COVID-19, the emergence of SARS-CoV-2 mutations, and the lack of definitive treatment, vaccination has become a priority of governments as the only way to control the disease [7,8]. Initially, the vaccination criteria varied across countries; for example, Austria and the United Kingdom prioritized people over 80 years old in the first phase of vaccination, while Germany and Estonia prioritized those over 70; in contrast, in Portugal, the age was 50 years, with priority assigned to those with a chronic disease, and, in Sweden, priority was given to all adults over 18 [9]. However, after the initial implementation of vaccinations, discrepancies were observed in the rate of coverage across countries. Despite the introduction of strategies and campaign vaccinations in all countries, the level of immunization as of 8 February 2021 in the European Union (EU) was below 5%, which was far from what was achieved in Israel (around 62%) [10,11]. In order to vaccinate a large amount of the population in an effective way, it is important to tackle organizational issues; however, it is also essential for the supply of vaccinations to be uninterrupted and unlimited, which, unfortunately, was not achieved in EU countries. The lack of fluidity in vaccine delivery significantly disrupted the smooth implementation of vaccination programs, further aggravating the differences in vaccination coverage between individual countries in the EU. Israel’s strategy made vaccinating the population a priority, put particular emphasis on organizational issues, and allocated all resources toward vaccinating the entire population as soon as possible. These factors put Israel in a privileged position in terms of obtaining vaccines during supply shortages.

When introducing a new vaccine (whether during a pandemic or as long-term prevention), three basic factors must be taken into account: (i) the structure, organization, and strength of the local healthcare system, (ii) the strength of government funding, and (iii) education of both vaccinators and vaccinated persons, with appropriate cultural adaptation [12]. However, during a pandemic, it is important to achieve high vaccination coverage as quickly as possible, thus preventing the virus from mutating. Therefore, there is no time to educate HCWs, teachers, parents, and children through formal meetings, as in the case of vaccines against hepatitis B or HPV, for example, as this can take years. In addition, for such diseases, it is necessary to maintain long-term vaccination continuity and a high level of coverage in the target group, and, in contrast to vaccinations against SARS-CoV-2, the personal and social effects of the vaccination campaign will not be noticeable until decades later [13,14].

On the other hand, even when a vaccine is widely available, it could be still a challenge to achieve population immunity, as pointed out by the World Health Organization (WHO) even before the COVID-19 pandemic. Although there is currently little doubt that vaccination is an effective approach to preventing infection and reducing the mortality of many infectious diseases, the WHO still classifies spreading hesitation and refusal to vaccinate as a global health threat. The WHO lists three main factors influencing vaccine hesitancy in the population: lack of access to vaccines, lack of confidence in and fear of vaccines, and underestimation of disease severity (lack of information about morbidity, complications after disease, and mortality) [15]. The best example of vaccine hesitancy is the low global level of influenza vaccine coverage, even among HCWs [16]. Therefore, in addition to ensuring vaccine availability and good vaccination program organization, there is a need for informational campaigns intended to raise awareness of the purpose and benefits of mass vaccination during a pandemic; their absence may result in the growth of conspiracy theories and skepticism against vaccination, which can build anti-vaccine movements [17].

The selection of the HCW priority group was important not only from an epidemic point of view, but also for educational purposes for those without access to reliable information about the vaccine. Because of the novel nature of the SARS-CoV-2 vaccine, the priority group (including HCWs) was burdened with the responsibility of relaying such information to the rest of society. Hence, the level of vaccination among HCWs and their acceptance of the vaccine could influence the level of vaccination among other social groups [18].

Apart from demographic factors, key factors in vaccine acceptance among HCWs seem to be personal experience related to COVID-19 and vaccination, the perception of oneself within a high-risk group, and working with patients suffering from COVID-19.

Therefore, this study examines how the decision to vaccinate against SARS-CoV-2 was influenced by the following factors: perceived risk of exposure to SARS-CoV-2, previous COVID-19 infection and the severity of symptoms, and the occurrence of COVID-19 disease in family and relatives. The aim of this retrospective research was also to assess the effectiveness of the organization the COVID-19 vaccination program in Poland and the relationship between the government campaign on vaccination and general attitudes to vaccination. In addition, the study evaluated trust in different sources of knowledge about the COVID-19 pandemic and vaccines against SARS-CoV-2 as predictors of acceptance of vaccination.

## 2. Materials and Methods

### 2.1. Participants

This retrospective study was conducted among 1200 health professionals, including salaried (doctors, nurses, and midwives) and non-salaried healthcare workers (medical, nursing, and midwifery students), in the Lodz voivodeship. The participants were recruited from one of the largest academic cities, situated in the center of Poland. With a target population of 6306 HCWs, a sample of at least 384 people should have been enrolled, within a 95% confidence level and a 5% margin of error [19]. Each of these two study groups included two subgroups. The HCWs consisted of junior participants, i.e., without completion of specialization (under 10 years of service) and senior participants, i.e., those with a specialization (over 10 years work experience). The non-salaried HCWs included junior students, i.e., attending mostly theoretical classes (from the lower years of study) and senior students, i.e., attending mostly clinical classes with patients (from higher years of study).

### 2.2. Questionnaire Survey and Study Design

The questionnaire consisted of 27 questions, but only 18 are discussed in this paper. These 18 questions were divided into the following categories: (i) demographic data, including age, gender, occupation, place of work, seniority, and coverage rate of COVID-19 vaccination; (ii) previous COVID-19 experience and severity of the disease (respondent and family); (iii) COVID-19 risk and anxiety; (iv) the impact of the government vaccination campaign on the decision to vaccinate, and the reliability of the different sources of information about COVID-19 and COVID-19 vaccinations (Supplementary Materials Table S1). The reliability of the different sources offering information about COVID-19 vaccinations was assessed using the following five-point Likert scale: very reliable (5 points), reliable (4 points), I do not know (3 points), not very reliable (2 points), and unreliable (1 point) [20].

The original questionnaire was designed on the basis of previous studies and the opinions of independent experts. A two-stage pre-study was carried out involving a total of 100 randomly selected HCWs 1 month before the main study. In the first stage of the pilot study, the participants evaluated each question according to whether it was clear enough and necessary for the final version of the questionnaire. In the second stage, the questionnaire was validated by standard procedures [19,21].

The study itself was conducted among HCWs at several Medical centers and the largest medical university in central Poland (including non-salaried HCWs, i.e., medical students) from 15 May to 15 July 2021. The target population comprised those who were working at that time or, in the case of students, attending classroom activities, so that they could complete the paper questionnaire. At that time, lockdown restrictions had been eased and practical classes were held at the medical university. No limiting factors were introduced, and the use of a paper-based questionnaire instead of an internet-based questionnaire guaranteed that the respondents belonged to the selected study group. The questionnaires were collected over 2 months in order to have contact with numerous respondents and allowing those who were temporarily absent from the university, e.g., due to illness or quarantine, to also complete the questionnaire.

### 2.3. Statistical Analysis

The different occupational groups and the vaccinated/unvaccinated groups were compared using proportions and *z*-tests for proportion. Results were marked with an asterisk if not all *z*-test assumptions were met. Simple (crude) and multiple logistic regression were run to estimate the odds ratio. As the *p*-value for the crude OR estimator is the same as that for the chi-square test for a two-way cross table, the results were interpreted in the context of odds ratios, as well as independence between groups. Data pretreatment was performed in Microsoft Excel, and statistical tests were carried out in STATISTICA 13.3 (Krakow, Poland) and GRETL (open-source statistical package).

### 2.4. Ethical Concerns

Before collecting the survey, the respondents were informed that their responses would be confidential and that the obtained data would be used only for scientific research. The questionnaire collected no identifying personal data from the participants. The study was performed in accordance with the Declaration of Helsinki or comparable ethical standards. According to Polish law and Good Clinical Practice regulations, the research, being an anonymous voluntary survey, did not require any approval from the Bioethics Committee [19].

## 3. Results

### 3.1. Sociodemographic Characteristics

Among 1200 initial participants, 1080 completed the paper version of the questionnaire; this group comprised 830 women (76.9%) and 250 men (23.1%). Regarding profession, the group consisted of 135 doctors (various specialties), 128 nurses and midwives, 423 medical students, and 394 students of nursing and midwifery. The mean age of the subjects was 26.8 years ± 9.7. The mean age of the nurses (N) was higher than that of the doctors (D) (41.4 ± 13.1; 38.3 ± 10.6 respectively); however, medical students (MS) and nursing and midwifery students (NS) were of similar age (23.6 ± 2.2 and 21.5 ± 2.1 years, respectively). In all subgroups, more women than men participated: 52.6% for D, 94.5% for N, 62.4% for MS, and 94.9% for NS. About 20% of the respondents declared that they had chronic diseases. The main workplace for working subjects was the hospital, while most of the students had not begun working yet. The sociodemographic data of the subjects are presented in Supplementary Materials Figure S1.

Most of the participants in our study were vaccinated (91.2%): 94.8% D, 78.9% N, 98.3% MS, and 86.3% NS (Supplementary Materials Figure S1). Among the unvaccinated participants, two subgroups were distinguished: those who have not yet been vaccinated but declared that they intend to do so, and those who did not want to be vaccinated at all (NWV—not wanting to vaccinate). The latter included 57 participants, including two D (1.5% of all D), 15 N (11.7% of N), three MS (0.7% of MS), and 37 NS (9.4% of NS).

### 3.2. COVID Risk Factor among HCWs

The strongest awareness of the high risk of COVID-19 infection was connected with occupation: the highest risk was noted for doctors (72.6%), followed by nurses and midwives (64.8%) (Table 1); these values are twice as high as those given by students. Almost half of the students did not identify with any risk group. Significant differences were noted between most groups with regard to self-perception as a high-risk group of COVID-19: D vs. MS (*p* < 0.001), D vs. NS (*p* < 0.001), N vs. MS (*p* < 0.001), N vs. NS (*p* < 0.001), and NS vs. MS (*p* = 0.025), but not between D and N (Table 1). About 15% of the respondents assigned themselves to the special risk group due to working in a crowded environment, while 5% assigned themselves due to chronic diseases (Table 1).

Feeling more stress than usual at work (because of COVID-19) was reported by 69.2% D and 60.2% N; about 30% considered this increase to be significantly higher (Table 1). Only 44.1% MS and 51.3% NS noted an increase in stress levels during the pandemic, as did approximately 50% of non-salaried HCWs (i.e., medical university students), regardless of the field of study. No statistically significant differences in stress levels were found between D and N (*p* = 0.445), but a significant increase was noted between the D group and students (MS *p* < 0.001 and NS *p* = 0.002), between N and MS (*p* < 0.001), and between MS and NS (*p* = 0.001).

In any case, where COVID-19 infection is suspected, a test is performed to confirm or deny SARS-CoV-2 infection. During the 6 months preceding the survey, fewer than 20% of D and N reported not being tested for SARS-CoV-2 at all, while approximately 50% of employees had to undergo the test more than three times (Table 1). In contrast, only 7–9% of students were repeatedly tested, and about 60% never needed a test. D and N were at a significantly high risk of infection and, thus, completed a higher frequency of tests, due to their professional activity; as such, significant differences were noted between D and MS, between D and NS, between N and MS, and between N and NS in this regard (*p* < 0.001; chi-square test).

### 3.3. Getting Sick with COVID-19 and the Course of COVID-19 among HCWs and Their Relatives

It was found that nurses and midwives (42.9%; confirmed with real-time RT-PCR) suffered from COVID-19 the most often, followed by doctors (33.3%). The students fell ill much less frequently (Table 2). Additionally, among the HCWs who contracted COVID-19, 25.1% decided to get vaccinated and 41.1% did not (*p* < 0.001). In the study, respondents who contracted COVID-19 were asked about their experience with the disease. In total, only six people were hospitalized due to COVID-19, including two who required ventilation for extra oxygen. A serious course of infection (with or without hospitalization) was reported more than twice as often in the case of D and N than by students (Table 2). Among the 57 respondents who developed serious COVID-19 illness without hospitalization, 20.5% decided to get vaccinated, and 15.4% did not (*p* = 0.458). All occupational groups, except medical students, reported no difference in the willingness to vaccinate due to previous COVID-19 infection. In the study population, 139 respondents reported the death of a family member and/or friend due to COVID-19. Additionally, 189 reported that they had relatives who had serious symptoms of the disease, and 145 reported that they had relatives who experienced serious complications (Table 2). However, only the death of relatives or serious disease symptoms were associated with an increased tendency to vaccinate, although the observed differences were not statistically significant (*p* = 0.066 and *p* = 0.051, respectively).

### 3.4. National SARS-CoV-2 Vaccine Program and Influence on COVID-19 Vaccination Decision

Of the 1074 people who answered the question about the impact of the government campaign on the decision to vaccinate, only a small percentage admitted that their immunization was significantly related to the vaccination campaign (6.7%) (Table 3); in contrast, almost twice as many considered the information provided in the campaign to be unreliable. More than half of the surveyed group of doctors and medical students showed no interest in the vaccination campaign, but they expressed a desire to get vaccinated whenever possible without any extra incentive (54.8% and 53.7%, respectively). Such declarations were made by fewer than one-third of N and NS (Table 3). About 30% of D, N, and MS and about 50% of NS were not interested in the campaign or did not see the campaign as an important source of information for their decision to vaccinate.

### 3.5. Types of Information Sources on Vaccination and SARS-CoV-2 Vaccines among HCWs

Regarding the source of information about vaccinations among the four study groups, D and MS generally preferred to base their vaccine-related information primarily on lectures and academic literature (almost 40%), N preferred the opinion of health professionals (approximately 40%), and NS (approximately 30%) preferred information from health professionals and websites (Table 3).

Among potential sources of information, academic lectures and literature were evaluated as the most reliable by all surveyed HCWs (Table 3). Health professionals were also highly rated as a reliable source of information on COVID-19 and vaccination. On the other hand, social media and information from friends were considered the least credible by all groups. Significant differences were found between occupational groups (D + MS and N + NS) regarding the source of pandemic- and vaccine-related information; D and MS were twice as likely to obtain knowledge from scientific sources as N and NS (39.3% vs. 19.9%) (*p* < 0.001) (Supplementary Materials Table S2), while the opposite was true for all other sources of information. However, the only significant difference was noted for traditional media, which nurses reported using more than twice as often as doctors (10.2% vs. 4.4%).

The multivariate logistic regression analysis showed that N and NS had lower chances of being vaccinated than D and MS (OR: 0.07 95% CI (0.02–0.18), *p* < 0.001) (Supplementary Materials Table S3). These comparisons were based on the group of vaccinated subjects and those who were reluctant to vaccinate (i.e., the NWV group). The group that had not yet been vaccinated but declared that they intended to were excluded from this analysis due to their uncertain vaccination views. Exposure to COVID-19 in the workplace, perceived exposure, or the number of tests performed previously indicating the possibility of infection had no significant influence (Supplementary Materials Tables S3 and S4).

The same logistic regression analysis found the following factors to influence vaccination coverage: (i) suffered from COVID; (ii) felt more stressed because of COVID-19; (iii) got information about the vaccination from radio, television, or press (Supplementary Materials Table S4). The multivariate logistic regression analysis found a higher probability of vaccination among those who suffered from COVID with mild symptoms than those with asymptomatic COVID-19 (OR: 7.58; 95% CI (1.86–30.93), *p* = 0.005); in addition, those who had experienced the death of relatives due to COVID-19 had a higher chance of getting vaccinated against SARS-CoV-2 than people who had not (OR: 4.45; 95% CI (1.00–19.83), *p* = 0.051) (Supplementary Materials Table S4). Moreover, it was found that vaccinated respondents significantly more often supported the obligation to vaccinate against COVID-19 among health professionals (OR: 0.14; 95% CI (0.06–0.29), *p* < 0.001) (Supplementary Materials Table S4).

**Figure 1:**
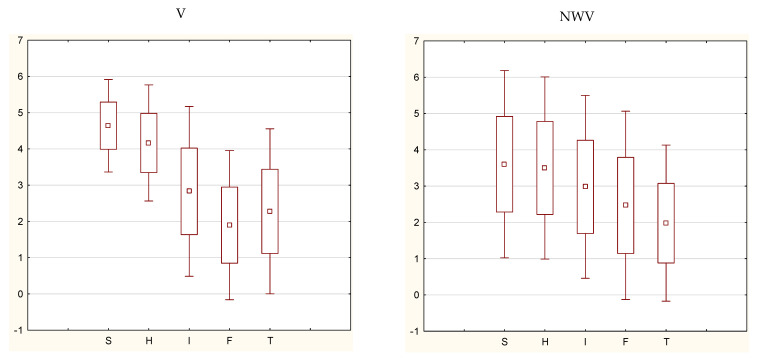
Reliability of individual information sources. Reliability of information sources: S—scientific source (academic lectures, medical literature, conferences, other courses), H—health professionals, I—internet sites dedicated to COVID-19, F—friends and social media, T—traditional media (radio, television, press); V—vaccinated, NWV—not wanting to vaccinate (mean, standard deviation, 1.96 × standard deviation).

Significant differences were found between vaccinated persons and vaccine-hesitant respondents regarding their assessment of reliable sources of information on vaccines and pandemics. The data were recorded on a five-point Likert scale (Figure 1).

Scientific sources had significantly higher credibility among vaccinated participants (mean credibility 4.64) than among NWV (mean credibility 3.60) (*p* < 0.001). Furthermore, health professionals were a significantly higher source of credibility among V (4.17) than among NVW (3.5) (*p <* 0.001), while friends and social networks had significantly lower credibility among V (1.90) than among NWV (2.5) (*p <* 0.001).

The differences in the assessment of the credibility of information sources between V and NWV are presented in Supplementary Materials Table S4. As these results were difficult to interpret in the OR category, a separate analysis for them was performed (Table 4). Vaccinated respondents considered the internet (32.3%) the least reliable source, but this was the case in only 20.0% of NWV (*p* = 0.015). On the other hand, only 2.5% of vaccinated HCWs found lectures and scientific literature unreliable; this value was five times higher among NWV people (*p <* 0.001). The vaccinated persons rarely indicated that information obtained from health professionals was unreliable (5.7%); this value was twice as high among the NWV respondents (*p <* 0.001). Generally, scientific sources (36.5% and 29.4%) and health professionals (34.4% and 30.3%) were seen as reliable sources of information for both groups (V and NWV). However, it was significantly more likely that the NWV group members would assess social media as credible compared to vaccinated respondents (*p* < 0.001). The same was true for knowledge about COVID-19 obtained from the internet (*p* = 0.038). Overall, NWV people were more skeptical about research sources and other healthcare professionals, and they considered social media and the internet as more reliable compared to respondents who got vaccinated (Table 4).

Interesting results were obtained regarding the impact of the government campaign by comparing the vaccinated groups with the NWV group (Table 5). It was found that the vaccinated groups were much more likely to find the government campaign unreliable than those who did not want to vaccinate.

## 4. Discussion

Over 1 year since the declaration of the novel coronavirus disease pandemic and 6 months after the launch of the SARS-CoV-2 National Vaccination Program [22], the present study set out to evaluate the organization of vaccination campaigns and the influence of information on COVID-19 vaccination level among health professionals. It analyzed the trust in different sources of knowledge about the COVID-19 pandemic and vaccines against SARS-CoV-2 as predictors of hesitancy or acceptance of vaccination. Assuming that attitudes toward vaccination may be related to experiences with COVID-19 (either personal experience or that of a relative), the study evaluated the role of perception of being in an “at-risk group” as a predictor for acceptance of vaccination against SARS-CoV-2.

A key benefit of this study was that the participants in “group zero” were able to take full advantage of the SARS-CoV-2 vaccinations before taking part in this study. Moreover, due to the duration of the pandemic, the respondents had already formed opinions about the pandemic itself and the available vaccines on the basis of different sources of information and experiences from their close professional and private environment.

From the first day of the vaccination campaign, all health professionals were eligible to receive the vaccine against SARS-CoV-2. The vaccine was free of charge and voluntary. Our findings indicated higher COVID-19 vaccine coverage among doctors and medical students (over 95%) than among nurses (over 78%) and students of nursing and midwifery (86%). In this study, the total percentage of HCWs in Poland vaccinated with all necessary doses was higher than in Spain at the same time (64%), with a similar population size. However, a similar percentage of HCWs in Poland refused to be vaccinated against SARS-CoV-2 (5.3%) compared to Spain (2.1%) [23] or Belgium (4.9%) [24], with some remaining undecided (3.5%).

Thus, the widespread availability of the SARS-CoV-2 vaccine did not result in the end of the pandemic, due to fluctuations in vaccination and the growing skepticism about vaccination, especially in the general population [25]. The vaccination coverage in the general population is usually significantly lower than in HCWs, and the attitudes toward COVID-19 vaccination in the general population must be assessed much more broadly than among HCWs, although some studies indicated some similarities in factors associated with vaccine reluctance in the general population and among medical staff [24]. The ability to develop and coordinate a vaccination campaign and broadcast truthful information is key to obtaining herd immunity, which is estimated by public health experts as immunization rates of over 70%, especially when vaccination has to be repeated several times. The high effectiveness of vaccination promotion campaigns is connected with understanding and overcoming hesitancy causes rather than persuading the convinced or strong refusers. This was confirmed by our research, where the largest group of vaccinated people were those who got vaccinated regardless of the national campaign, signifying that people who have decided to vaccinate do not require any additional promotional campaigns. Thus, programs and information should be more targeted at those who are hesitant. Vaccine hesitancy is distinct from anti-vaccination ideology and can be overcome with the right strategies [26].

A crucial issue could also be the epidemic-induced sense of danger, which may influence the decision to vaccinate. Increasing stress levels among health professionals during the pandemic were related to both their work and their increased risk of COVID-19 disease, as well as the adoption of the vaccine against SARS-CoV-2, whose approval coincided with the commencement of mass vaccination without earlier proper information on adverse events. In our study, the highest stress was more often reported by doctors, followed by nurses, and then by students (*p* < 0.05). This is undoubtedly related to a much lower probability of exposure to COVID-19 for students, especially in the earlier study years, who did not have daily contact with patients. The frequency of exposure and, hence, the risk of falling ill was also evidenced by the frequency of tests for the presence of the SARS-CoV-2 virus; these were much more common among salaried HCWs (D and N) than non-salaried HCWs. In addition, active workers were slightly more likely to contract COVID-19 and develop more serious symptoms than students. On the other hand, contracting COVID-19 did not prove to be a sufficient premise for vaccination among the respondents who firmly refused to do so. The reason was most likely the asymptomatic course of the disease and the belief that infection is not dangerous. Meanwhile, being unconvinced about the scale and degree of risk of the COVID-19 pandemic and asymptomatic infections could be a crucial barrier to controlling the spread of SARS-CoV-2 infection [27]. Research has shown that asymptomatic patients are the cause of 40–45% of all infections.

Vaccination coverage was increased by death among relatives and the serious symptoms of the disease. Focusing on empathy and a civil duty to protect individuals at particular risk for COVID due to age or medical history should be especially important in the case of health professionals and their relationships with patients. Similarly, other studies noted that the death of a family member or friend due to COVID-19 increased the chance of getting vaccinated [28]. Additionally, other studies revealed that contact with people who suffered from COVID-19 [29] led to higher vaccine acceptance than employer pressure [30].

Excluding the first months, when the availability of vaccines in Europe was limited, the anti-COVID-19 immunization program in Poland was very well organized. The most important factor was easy access to vaccines. The infrastructure for vaccination availability was significantly expanded. Numerous vaccination points were created, making it possible to get vaccinated not only in outpatient clinics but also at the workplace or school, while people with disabilities and seniors were vaccinated at their place of residence. Eventually, due to legislative changes, pharmacists also obtained permits for vaccination. It was enough to report the desire to vaccinate by e-mail or by phone to be registered free of charge, indicate a preference for time and place, and then appear at a selected point. In Poland, similar to Hungary and Greece [31], registration was required, which introduced order and prevented possible unfair abuse. At the time of the pandemic’s decrease (fewer COVID-19 cases and deaths), the campaign had to introduce increasing incentives to vaccinate by offering citizens the chance to participate in the lottery and the possibility to win cash money or a prize upon receiving the vaccine. We are not aware of any studies on the impact of the vaccine national lottery in Poland on the desire to be vaccinated, but the COVID-19 vaccine lotteries were found to increase vaccine immunization coverage in the US [32,33]. This form of incentive to vaccinate may seem particularly desirable during the pandemic when it is important to achieve herd immunity as soon as possible, and the prolonged state of an epidemiological threat can reduce motivation.

Unfortunately, the national vaccination agenda in Poland did not prove successful among vaccine-hesitant people. When reviewing the national vaccine rollout, it seems that mandating vaccination or placing restrictions on the enjoyment of cultural and social life among the unvaccinated may appear to be an easier strategy to increase vaccine uptake than developing special communication efforts. However, it is a strategy with the potential to backfire, because the perception of the coercive effect is highly dependent on the country’s culture. A study conducted in Hong Kong indicated that the government’s recommendations were the strongest predictor of vaccination [34], while a survey among HCWs in the UK realized that higher pressure to get vaccinated among healthcare professionals increased the likelihood of rejecting the COVID-19 vaccine [30]. Furthermore, in line with earlier Polish analyses, overtly authoritarian decisions by the government were overall met with a negative social response [35]. Moreover, because the campaign is aimed primarily at the distrustful, it should draw on authorities who are commonly considered trustworthy. Incorporating a community voice could help build vaccine confidence among this population [26,36]. Higher importance should be also given to indirect campaigns, because this group tends to place greater trust on family and friends.

Currently, hesitant populations are primarily those who fear the adverse reaction of the vaccines more than COVID-19 diseases [17,37]. When shaping campaign strategies, these factors need to be addressed and countered by spreading validated information about pandemics and vaccination. This is especially important when studying vaccine efforts in Poland, as, regardless of changes in the intensity of the pandemic, the percentage of people who are skeptical about vaccination remains high [38]. The factor that played a significant role in reducing confidence in vaccination in Poland was the inconsistencies in the opinions presented in the campaign. The disputes on the value of the vaccine, the necessity to use it, the risks of its use, and public rejection of vaccination by some politicians contradicted what representatives of the ruling camp conveyed as part of the campaign and diminished its credibility. Meanwhile, the model shows that, in the absence of sufficient knowledge to assess which attitude is wrong, a typical person will most likely take the safest solution, i.e., distrust toward both positions [39]. Moreover, according to omission bias, individuals prefer taking a passive risk (i.e., not vaccinating) than taking a risk through active behavior [40]. Therefore, it is essential that the information provided in the campaign and other sources, including by scientists and experts, is consistent. Indeed, analyses of the economic aspect of the vaccination programs, which cannot be disregarded, have shown that large-scale vaccination programs are profitable. National vaccination campaigns require appropriate funds for many things, including the purchase of the vaccine by the state and the logistical organization of the vaccination effort. An economic evaluation of the mass vaccination effort based on the three most popular COVID-19 vaccines (Pfizer/BioNTech, Moderna, and Oxford/AstraZeneca) found them to be highly effective [41]. A cost–benefit analysis of these vaccines found that 1 USD invested in the vaccine would save 13 USD, 23 USD, and 28 USD, respectively, in the case of no vaccination, when health and education loss were considered. The corresponding figures taking the value of the statistical life into account were 176 USD, 300 USD, and 443 USD, respectively [41].

Vaccine acceptance is a complex problem that reflects not just the acceptance of a pharmaceutical product, but also the ideologies that are used for its distribution [17]. The rush to roll out the COVID-19 vaccinations resulted in disorganized public health communications which dissuaded some undecided from vaccination. Having concerns about the potential side-effects of the COVID-19 vaccine is valid, and not considering the legitimacy and importance of these concerns is a mistake. A successful communication strategy for addressing mistrust is to share knowledge through relatable sources. Educational campaigns may be effective means for addressing knowledge gaps and correcting misinformation. However, due to the great diversity of the target group, it seems that universal programs may be ineffective, and adapting the communication to individual populations is important. This may also partially explain why pro-vaccine campaigns in Poland were of very limited success. The national vaccination agenda was focused on reaching the widest possible populations in the shortest possible time and did not appropriately adapt to follow the specific social groups that were to be vaccinated. Moreover, vaccine information should be conveyed in a personalized way so that it is understandable and needed for a given group of recipients [42]. The Centers for Disease Control and Prevention [43] also emphasizes the importance of using everyday terminology in public health campaigns targeting the general population.

In communicating the available knowledge to the public, it is also important to maintain a balance between the speed of disclosure of information and its accuracy and reliability. One should also emphasize verifying the source of the information. Despite the common knowledge that social networks prefer sensation over accuracy, a large proportion of the general population browses websites every day. As our research shows, healthcare professionals primarily assessed scientific sources as reliable and were able to largely assess the reliability of the information they provided. As such, most of them, regardless of the course of the national vaccination campaign, had already made the decision to vaccinate. A large proportion of the respondents, however, did not have a positive opinion about the vaccination program as a source of information, and this may be of great importance when it comes to understanding the attitude of the general population. The limited accuracy of the information on the internet is often a result of the fact that anyone can potentially publish health information [44]. Moreover, internet sources are often blamed for the rise in vaccine skepticism because of their role in spreading misinformation on an unprecedented scale, which is used by anti-vaccination movements. It is very difficult to limit this phenomenon as long as the internet is the main source of information about health problems in the general population. Meanwhile, the more people trust the internet as a source of health knowledge, the less positive their attitudes are toward vaccination; people who search the internet for information often end up with arguments spread by anti-vaccine activists [44]. Furthermore, this group is also more likely to have limited trust in HCWs and specialists. Meanwhile, evidence from previous studies showed that trust in physicians and science increased in some countries during the pandemic, but decreased in others [45]; while the first trend is clearly conducive to a positive attitude toward vaccinations, the latter is associated with higher skepticism [44].

### Limitations of This Study

The main limitation of the study was the fact that most of the respondents who participated decided to get vaccinated (91.2%); hence, there were large differences in the size of the groups when comparing the motivations between the vaccinated and the unvaccinated HCWs. Moreover, the research group was hermetic and included only people with higher medical education, which could be expected to have a positive approach to vaccination.

## 5. Conclusions

The study investigated attitudes among health professionals toward vaccination after considering their own experiences, the experiences of their relatives with the disease, opinions on the credibility of available sources of information, and the national vaccination campaign as factors encouraging or discouraging vaccination against SARS-CoV-2. Achieving herd immunity with sufficient vaccine availability requires encouragement among a large proportion of the population. To achieve this goal, it is necessary to identify hesitant groups and identify the reasons for their attitudes. Well-planned public health campaigns should focus on the causes of hesitation before immunization. An important element is the creation of appropriate communication strategies for targeting different populations to properly educate individuals on the pandemic, the vaccine, and the consequences that come with refusing a vaccination.

## Figures and Tables

**Table 1:** Risk assessment of morbidity and the stress associated with it.

	D	N	MS	NS	Total	D vs. N	D vs. MS.	D vs. NS	N vs. MS	N vs. NS	NS vs. MS
High risk of getting COVID-19 (multiple choice)											
Yes, as I am a health service employee	119 (72.6%)	105 (64.8%)	158 (36.1%)	116 (28.8%)	498 (42.7%)	0.132	<0.001	<0.001	<0.001	<0.001	0.025
Yes, as I work in a large group of people	19 (11.6%)	24 (14.8%)	50 (11.4%)	73 (18.1%)	166 (14.2%)	0.390	0.954	0.056	0.261	0.347	0.006
Yes, as I have chronic illnesses	8 (4.9%)	13 (8.0%)	17 (3.9%)	23 (5.7%)	61 (5.2%)	0.248 *	0.585 *	0.694 *	0.039	0.308	0.214
Yes, because of age	11 (6.7%)	9 (5.6%)	2 (0.5%)	1 (0.2%)	23 (2.0%)	0.665 *	<0.001 *	<0.001 *	<0.001 *	<0.001 *	0.613 *
No	7 (4.3%)	11 (6.8%)	211 (48.2%)	190 (47.1%)	419 (35.9%)	0.320 *	<0.001 *	<0.001 *	<0.001	<0.001	0.766
Total	164 (100.0%)	162 (100.0%)	438 (100.0%)	403 (100.0%)	1167 (100.0%)						
Higher stress due to COVID-19											
No, it does not matter to me	20 (15.0%)	21 (16.4%)	141 (33.4%)	108 (27.5%)	290 (27%)	0.762	<0.001	0.004	<0.001	0.012	0.070
No, but I am always stressed	21 (15.8%)	30 (23.4%)	95 (22.5%)	83 (21.2%)	229 (21.3%)	0.121	0.097	0.179	0.827	0.590	0.645
Yes, a little higher	64 (48.1%)	53 (41.4%)	161 (38.2%)	151 (38.5%)	429 (39.9%)	0.277	0.042	0.052	0.509	0.562	0.914
Definitely yes, much higher	28 (21.1%)	24 (18.8%)	25 (5.9%)	50 (12.8%)	127 (11.8%)	0.642	<0.001	0.020	<0.001	0.092	0.001
Total	133 (100.0%)	128 (100.0%)	422 (100.0%)	392 (100.0%)	1075 (100.0%)						
Were you tested for COVID-19 due to risk of contamination?											
Yes, many times	74 (54.8%)	59 (46.1%)	30 (7.1%)	35 (8.9%)	198 (18.4%)	0.159	<0.001	<0.001	<0.001	<0.001	0.349
Yes, several times (2–3)	28 (20.7%)	29 (22.7%)	61 (14.5%)	39 (9.9%)	157 (14.6%)	0.707	0.087	0.001	0.030	<0.001	0.048
Yes, once	16 (11.9%)	16 (12.5%)	82 (19.5%)	65 (16.6%)	179 (16.7%)	0.872	0.042	0.189	0.070	0.269	0.277
No	17 (12.6%)	24 (18.8%)	247 (58.8%)	253 (64.5%)	541 (50.3%)	0.170	<0.001	<0.001	<0.001	<0.001	0.094
Total	135 (100.0%)	128 (100.0%)	420 (100.0%)	392 (100.0%)	1075 (100.0%)						

D—doctors, N—nurses and midwives, MS—medical students, NS—nursing and midwifery students; * low-credibility data due to the low size of the compared groups (note that the result for the *z*-test for two proportions gives the same *p*-value as chi-square test of independence for which the sample size assumption is weaker).

**Table 2:** Incidence and symptoms of COVID-19 among respondents and their relatives.

	D 135 (12.5%)		*p*	N 128 (11.8%)		*p*	MS 423 (39.2%)		*p*	NS 394 (36.5%)		*p*	Total 1080 (100%)		*p*
	V (128)	NV (7)		V (101)	NV (27)		V (416)	NV (7)		V (340)	NV (54)		V (985)	NV (95)	
Were you ill with COVID-19?															
Yes	42 (32.8%)	3 (42.9%)	0.583 *	43 (42.6%)	12 (44.4%)	0.862 *	87 (20.9%)	5 (71.4%)	0.001 *	75 (22.1%)	19 (35.2%)	0.036	247 (25.1%)	39 (41.1%)	0.001
No	86 (67.2%)	4 (57.1%)	0.583 *	58 (57.4%)	15 (55.6%)	0.862 *	329 (79.1%)	2 (28.6%)	0.001 *	265 (77.9%)	35 (64.8%)	0.036	738 (74.9%)	56 (58.9%)	0.001
Symptoms of COVID-19															
Hospital with use of a life-supporting machine	0 (0.0%)	0 (0.0%)	-	1 (2.3%)	0 (0.0%)	0.596 *	0 (0.0%)	1 (20.0%)	<0.001 *	0 (0.0%)	0 (0.0%)	-	1 (0.4%)	1 (2.6%)	0.132 *
Hospital without use of a life-supporting machine	3 (7.1%)	0 (0.0%)	-	1 (2.3%)	0 (0.0%)	-	0 (0.0%)	0 (0.0%)	-	0 (0.0%)	0 (0.0%)	-	4 (1.6%)	0 (0.0%)	-
Serious illness without hospitalization	11 (26.2%)	1 (33.3%)	0.788 *	17 (39.5%)	1 (8.3%)	0.047 *	11 (12.6%)	1 (20.0%)	0.636 *	12 (16.0%)	3 (15.8%)	0.982 *	51 (20.7%)	6 (15.4%)	0.458 *
Mild infection	26 (61.9%)	1 (33.3%)	0.335 *	17 (39.5%)	6 (50.0%)	0.519 *	68 (78.2%)	3 (60.0%)	0.349 *	49 (65.3%)	11 (57.9%)	0.498 *	160 (64.8%)	21 (53.8%)	0.177 *
Asymptomatic infection	2 (4.8%)	1 (33.3%)	0.062 *	7 (16.3%)	5 (41.7%)	0.065 *	8 (9.2%)	0 (0.0%)	-	14 (18.7%)	5 (26.3%)	0.427 *	31 (12.6%)	11 (28.2%)	0.011
Total	42 (100.0%)	3 (100.0%)	-	43 (100.0%)	12 (100.0%)		87 (100.0%)	5 (100.0%)	-	75 (100.0%)	19 (100.0%)	-	247 (100.0%)	39 (100.0%)	
COVID disease in relatives (multiple choice)															
Death	26 (16.9%)	0 (0.0%)	-	17 (14.3%)	2 (6.5%)	0.245 *	47 (10.0%)	1 (14.3%)	0.707 *	43 (11.0%)	3 (5.1%)	0.163 *	133 (11.7%)	6 (5.8%)	0.066 *
Serious illness	27 (17.5%)	0 (0.0%)	-	20 (16.8%)	4 (12.9%)	0.598 *	70 (14.9%)	0 (0.0%)	-	63 (16.1%)	5 (8.5%)	0.127 *	180 (15.9%)	9 (8.7%)	0.051 *
Illness and post-illness complications	16 (10.4%)	1 (14.3%)	0.743 *	19 (16.0%)	6 (19.4%)	0.653 *	45 (9.6%)	0 (0.0%)	-	53 (13.6%)	5 (8.5%)	0.278 *	133 (11.7%)	12 (11.5%)	0.957
Illness, but no post-illness complications	58 (37.7%)	2 (28.6%)	0.627 *	39 (32.8%)	10 (32.3%)	0.957 *	205 (43.5%)	4 (57.1%)	0.471 *	137 (35.0%)	31 (52.5%)	0.010	439 (38.7%)	47 (45.2%)	0.193
No illness	27 (17.5%)	4 (57.1%)	0.010 *	24 (20.2%)	9 (29.0%)	0.290 *	104 (22.1%)	2 (28.6%)	0.682 *	95 (24.3%)	15 (25.4%)	0.851	250 (22.0%)	30 (28.8%)	0.112
Total	154 (100.0%)	7 (100.0%)		119 (100.0%)	31 (100.0%)		471 (100.0%)	7 (100.0%)		391 (100.0%)	59 (100.0%)		1135 (100.0%)	104 (100.0%)	

D—doctors, N—nurses and midwives, MS—medical students, NS—nursing and midwifery students, V—vaccinated, NV—non vaccinated. * Low-credibility data due to the low size of the compared groups; - no data in a given group for statistical analysis; *p*-value for *z*-test and chi-square test.

**Table 3:** Influence of the campaign and sources of obtained information.

	D	N	MS	NS	Total	D vs. N	D vs. MS.	D vs. NS	N vs. MS.	N vs. NS	NS vs. MS
Influence of government campaign on vaccination decision											
Definitely yes	12 (8.9%)	17 (13.6%)	24 (5.7%)	19 (4.9%)	72 (6.7%)	0.229	0.186	0.087	0.003	0.001	0.604
No, I was going to take the vaccine any way	74 (54.8%)	40 (32.0%)	227 (53.7%)	125 (32.0%)	466 (43.4%)	<0.001	0.815	<0.001	<0.001	0.995	<0.001
It did not matter to me	20 (14.8%)	13 (10.4%)	42 (9.9%)	56 (14.3%)	131 (12.2%)	0.286	0.116	0.888	0.878	0.263	0.055
No, I was not interested in this campaign	17 (12.6%)	28 (22.4%)	97 (22.9%)	140 (35.8%)	282 (26.3%)	0.038	0.010	<0.001	0.901	0.006	<0.001
Definitely no, the information was not too reliable in my opinion	12 (8.9%)	27 (21.6%)	33 (7.8%)	51 (13.0%)	123 (11.5%)	0.004	0.686	0.200	<0.001	0.020	0.014
Total	135 (100.0%)	125 (100.0%)	423 (100.0%)	391 (100.0%)	1074 (100.0%)						
Where did you usually get information about the vaccination (multiple choice)?											
Academic lectures, medical literature, etc.	88 (40.2%)	42 (22.5%)	251 (38.4%)	117 (19.4%)	498 (29.9%)	<0.001	0.647	<0.001	<0.001	0.357	<0.001
Health professionals	67 (30.6%)	76 (40.6%)	159 (24.3%)	181 (30.0%)	483 (29.0%)	0.035	0.068	<0.001	<0.001	0.007	0.025
The internet, sites dedicated to COVID-19	42 (19.2%)	43 (23.0%)	193 (29.6%)	203 (33.6%)	481 (28.9%)	0.347	0.003	0.005	0.079	0.006	0.122
Friends, as well as social media	7 (3.2%)	5 (2.7%)	25 (3.8%)	45 (7.5%)	82 (4.9%)	0.757 *	0.667 *	0.004 *	0.453 *	0.019 *	0.005
Radio, television, press	15 (6.8%)	21 (11.2%)	25 (3.8%)	58 (9.6%)	119 (7.2%)	0.122	0.065	0.229	<0.001	0.517	<0.001
Total	219 (100.0%)	187 (100.0%)	653 (100.0%)	604 (100.0%)	1663 (100.0%)						
How do you rate the reliability of the different sources information on a scale of 1–5 (where 1—unreliable, 2—not very reliable, 3—I do not know, 4—reliable, 5—very reliable)											
Academic lectures, medical literature, etc.	4.6 (0.7)	4.2 (0.5)	4.8 (0.8)	4.4 (0.7)	4.6 (0.0)	<0.001	0.036	0.002	<0.001	0.001	<0.001
Health professionals	4.0 (0.8)	4.0 (0.8)	4.1 (0.9)	4.2 (0.9)	4.1 (0.0)	0.902	0.089	0.004	0.072	0.003	0.097
The internet, sites dedicated to COVID-19	2.8 (1.2)	3.1 (1.2)	2.7 (1.2)	2.9 (1.2)	2.8 (0.0)	0.033	0.739	0.177	0.003	0.207	0.016
Friends, as well as social media	2.0 (1.1)	2.0 (0.9)	1.7 (1.2)	2.2 (1.1)	1.9 (0.0)	0.599	0.013	0.104	0.001	0.270	<0.001
Radio, television, press	2.4 (1.2)	2.4 (1.1)	2.1 (1.2)	2.4 (1.2)	2.3 (0.0)	0.629	0.019	0.911	0.002	0.622	<0.001

D—doctors, N—nurses and midwives, MS—medical students, NS—nursing and midwifery students. * Low-credibility data due to the low size of the compared groups.

**Table 4:** Reliability evaluation of given sources of vaccination knowledge.

	Reliable			Not Reliable		
	Vaccinated	Not Wanting to Vaccinate		Vaccinated	Not Wanting to Vaccinate	
	*n* (%)		*p*	*n* (%)		*p*
Scientific	894 (36.5%)	32 (29.4%)	0.131	34 (2.5%)	12 (13.3%)	<0.001
Health staff	844 (34.4%)	33 (30.3%)	0.377	77 (5.7%)	13 (14.4%)	<0.001
Internet	379 (15.5%)	25 (22.9%)	0.038	435 (32.3%)	18 (20.0%)	0.015
Friends and social media	123 (5.0%)	14 (12.8%)	<0.001	381 (28.3%)	23 (25.6%)	0.581
Traditional media	212 (8.6%)	5 (4.6%)	0.141	418 (31.1%)	24 (26.7%)	0.382
Total	2452 (100.0%)	109 (100.0%)		1345 (100%)	90 (100%)	

On a scale of 1–5 (where 1—unreliable, 2—not very reliable, 3—I do not know, 4—reliable, 5—very reliable); results of 1 + 2 were considered not reliable, while results of 4 + 5 were considered reliable.

**Table 5:** Influence of the government campaign on the decision to vaccinate.

	Vaccinated	Not Wanting to Vaccinate	Total	*p*
Yes.	68 (6.9%)	3 (5.4%)	71 (6.8%)	0.665 *
No, I was going to take the vaccine anyway.	459 (46.7%)	3 (5.4%)	462 (44.5%)	<0.001 *
It did not matter to me.	112 (11.4%)	10 (17.9%)	122 (11.7%)	0.142
I was not interested in this campaign.	246 (25.0%)	18 (32.1%)	264 (25.4%)	0.235
The information was not reliable in my opinion.	98 (10.0%)	22 (39.3%)	120 (11.5%)	<0.001
Total	983 (100.00%)	56 (100.0%)	1039 (100.0%)	

* Low-credibility data due to the low size of the compared groups.

## Data Availability

The dataset we analyzed for the current study is available from the corresponding author on request.

## Supplementary Materials

The following supporting information can be downloaded at https://www.mdpi.com/article/10.3390/ijerph19127231/s1, Table S1: The survey questionnaire; Table S2: Sources of information on COVID-19; Table S3: Associations between characteristics of subjects (sex, age, occupation, seniority, and exposure to COVID in workplace) and decision about vaccination; Table S4: Associations between possible decision-making factors and decision about vaccination; Figure S1: Sociodemographic characteristics of participants.
