# Supplementary material for: A Retrospective Cross-Sectional Study on the Risk of Getting Sick with COVID-19, the Course of the Disease, and the Impact of the National Vaccination Program against SARS-CoV-2 on Vaccination among Health Professionals in Poland
Source: Int J Environ Res Public Health. 2022 Jun 13;19(12):7231. doi: 10.3390/ijerph19127231 (PMC9223641; doi:10.3390/ijerph19127231)
Supplement: Supplementary file 1 [file ijerph-19-07231-s001.zip › ijerph-1728413-supplementary.pdf]

Figure S1. Sociodemographic characteristics of participants.

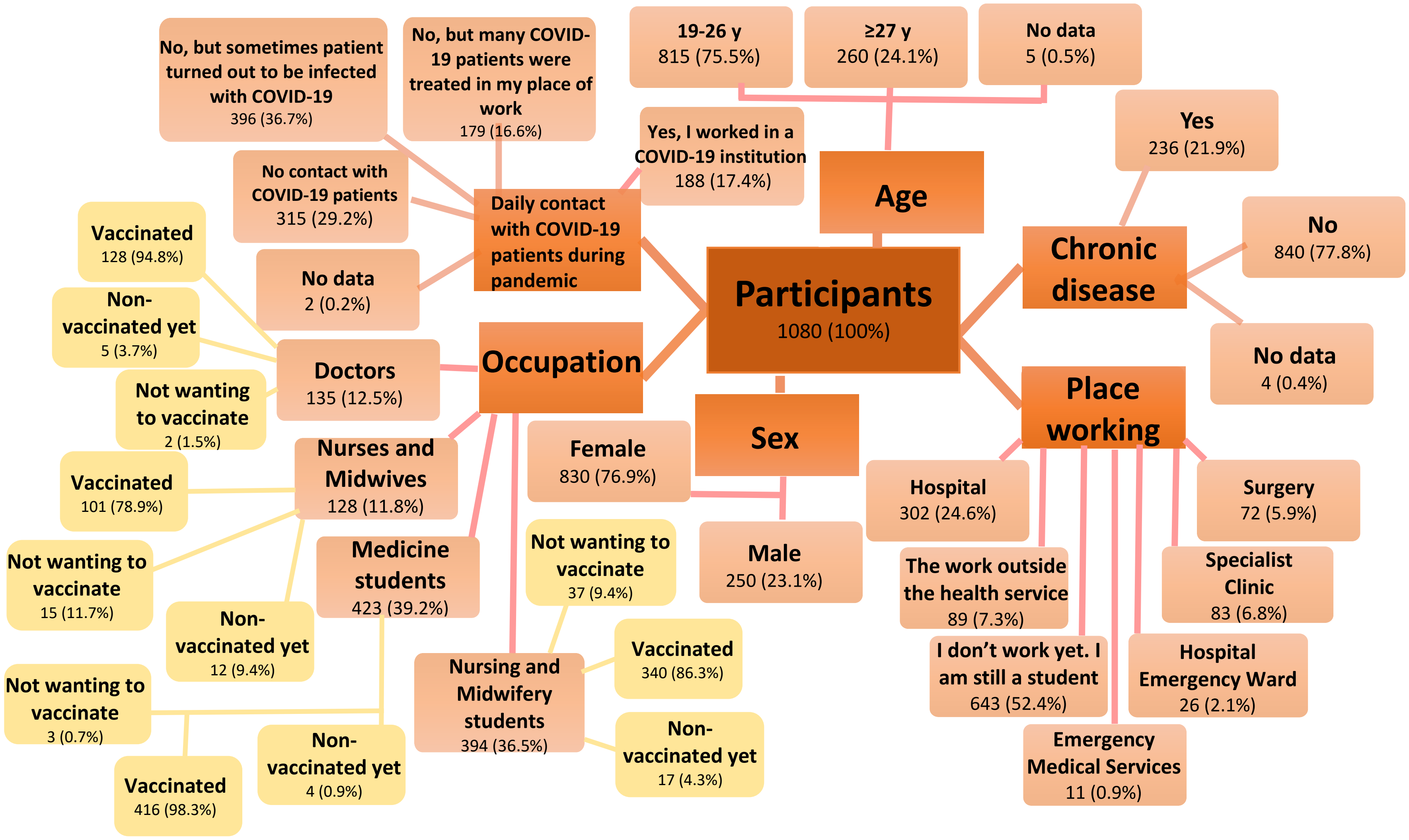

Table S1. The survey questionnaire.

Questions	Answers
Did you take the COVID-19 vaccine?	• Yes<br>• No, but I intend to take it in the near future<br>• No, I don't want to take the COVID-19 vaccine
Do you think you are in the group of a "high risk to get COVID-19"?	• Yes, as I am a health service employee<br>• Yes, as I work in a large group of people<br>• Yes, as I have chronic illnesses<br>• Yes, because of age<br>• No, I am not at risk
Did you feel more stressed doing your job (internship, classes at university) because of the COVID-19 pandemic?	• No, it didn't matter to me.<br>• No, but I was always stressed<br>• Yes, a little higher<br>• Definitely yes, the stress level was much higher than usual.
Were you tested on COVID-19 during the last year due to the possible contact with an infected person?	• Yes, many times (more than 3)<br>• Yes, several times (2-3)<br>• Yes, it happened once<br>• No.
Were you ill with COVID-19?	• Yes<br>• No
Because of COVID-19:	• I was in hospital and had to use the life-supporting machine<br>• I was in hospital, but there was no need to use the life-supporting machine<br>• I was seriously ill, but there was no need to go to hospital<br>• I had mild infection<br>• I didn't have any symptoms (asymptomatic infection)
Did any member of your family or any of your friends who caught the COVID-19 (more than one answer possible)	• died<br>• was seriously ill<br>• has or had complications<br>• was ill, but is OK now and back to their normal activity<br>• No, nobody among my family or friends had COVID-19
Did the Government Campaign for the National Vaccine Program have any influence on your decision to take the vaccine?	• Definitely yes<br>• No, I was going to take the vaccine any way<br>• It didn't matter to me<br>• No, I wasn't interested in this campaign<br>• Definitely no, the campaign wasn't very reliable in my opinion
Where did you usually get the information about the vaccination and the offered vaccines? (more than one answer possible)	• academic lectures, medical literature, conferences, other courses<br>• health professionals<br>• the Internet, the sites dedicated to COVID-19<br>• friends, also the social media<br>• radio, television, press<br>• others, please give an example…………..

How do you rate the reliability of the different sources offering information about vaccinations? (Please put an X next to the chosen answer).	• Very reliable (5)<br>• Reliable (4)<br>• I don't know (3)<br>• Not very reliable (2)<br>• Unreliable (1)
If the COVID-19 vaccination was recommended to be taken every year, should it be obligatory for the health professionals in your opinion?	• Yes, it should be obligatory<br>• Yes, it should be obligatory, if it is free of charge<br>• No, it should be voluntary
Sex	• Woman<br>• Man
Age	• in years ………………………………
Do you have any chronic diseases?	• Yes<br>• No
Occupation	• Nurse/ Midwife<br>• Doctor<br>• Nursing/ Midwifery student<br>• Medicine student
Job/ study seniority	• (in years)…………………
Place of work (you can chose several)	• Hospital<br>• Outpatient clinic<br>• Specialist clinic<br>• Hospital Emergency Ward<br>• Emergency<br>• The work outside the health service<br>• I don't work yet, I am still a student
Did you have daily contact with COVID-19 patients during the pandemic?	• Yes, I worked in an institution which is or temporarily was turned into a COVID-19 institution<br>• No, but many COVID-19 patients were treated in my place of work<br>• No, but it sometimes turned out later that the patient was infected with COVID-19<br>• No, I had no contact with COVID-19 patients

Table S2. Sources of information on COVID-19.

	**N and NS**	**D and MS**	***p***
Academic lectures, medical literature etc.	151 (19.9%)	338 (39.3%)	<0.001
Health professionals	244 (32.2%)	225 (26.2%)	0.008
The Internet, the sites dedicated to COVID-19	236(31.1%)	229 (26.7%)	0.051
Friends. also social media	47 (6.2%)	29 (3.4%)	0.008
Radio. television. press	77 (10.2%)	38 (4.4%)	<0.001
Total	758 (100%)	859 (100%)	

D- Doctors, N- Nurses and Midwives, MS – Medical Students, NS - Nursing and Midwifery students.

Table S3. Associations between characteristics of subjects (sex, age, occupation, seniority, exposure to COVID in workplace) and decision about vaccination.

	V	NWV	**Univariate Logistic Regression**		**Multivariate Logistic Regression**	
			OR 95%CI	*p*	OR 95%CI	*p*
			Sex			
Male	237	6	1.00 Ref.			
Female	748	51	0.37 (0.16-0.88)	0.019	1.51 (0.56-4.08)	0.418
			Age			
≥27 year	228	17	1.00 Ref.			
19-26 year	757	39	1.45 (0.80-2.61)	0.216	1.82 (0.88-3.74)	0.105
			Chronic disease			
Yes	211	16	1.00 Ref.			
No	774	41	0.70 (0.38-1.27)	0.237	0.73 (0.38-1.40)	0.347
			Occupation			
D+MS	441	5	1.00 Ref.			
N+NS	544	52	0.12 (0.05-0.30)	<0.001	0.07 (0.02-0.18)	<0.001
			Seniority			
Junior	341	27	1.00 Ref.			
Senior	644	30	1.70 (0.99-2.91)	0.050	1.10 (0.60-2.0)	0.749
			Exposure to COVID			
Yes, I worked in a COVID-19 institution	286	20	0.54 (0.27-1.09)	0.083	1.28 (0.54-3.05)	0.571
No, but many COVID-19 patients were treated in my place of work	168	13	0.49 (0.23-1.07)	0.067	1.72 (0.69-4.26)	0.245
No, but sometimes patient turned out to be infected with COVID-19	161	9	0.68 (0.29-1.60)	0.373	1.82 (0.87-3.84)	0.111
No contact with COVID-19 patients	369	14	1.00 Ref.			

D- Doctors, N- Nurses and Midwives , MS – Medical Students, NS - Nursing and Midwifery students; Junior- first years of study (1-3 for MS, 1 for NS) / up to 10 years inclusive of work in the profession; Senior- last years of study (4-6 years for MS, 2-3 years for NS) / more than 10 years of work in the profession; V-vaccinated, NWV – not wanting to vaccinated.

Table S4. Associations between possible decision-making factors and decision of vaccination.

	Vaccinated	Not wanting to vaccinate	Univariate Logistic Regression		Multivariate Logistic Regression	
			OR 95%CI	*p*	OR 95%CI	*p*
		High risk group				
Yes, a health service employee	467	19	1.62 (0.88-2.99)	0.118	1.24 (0.58-2.67)	0.581
Yes, work in a large group of people	149	11	0.89 (0.43-1.86)	0.764	0.54 (0.21-1.36)	0.190
Yes, chronic illnesses	55	2	1.81 (0.42-7.87)	0.420	0.99 (0.16-6.03)	0.993
Yes, because of age	19	2	0.63 (0.14-2.84)	0.541	1.34 (0.13-14.22)	0.807
No	379	25	1.00 Ref.			
		Suffered from COVID-19				
Yes	247	20	1.00 Ref.			
No	738	38	1.57 (0.90-2.75)	0.111	5.20 (1.61-16.74)	0.006
		Symptom of COVID-19				
I was in hospital or seriously ill without hospitalization	56	2	7.23 (1.44-36.17)	0.007*	5.59 (0.70-44.88)	0.105*
Mild infection	162	10	4.18 (1.53-11.43)	0.003	7.58 (1.86-30.93)	0.005
Asymptomatic infection	31	8	1.00 Ref.			
		COVID-19 disease of relatives				
Died	133	3	3.55 (1.03-12.15)	0.032	4.45 (1.00-19.83)	0.051
Seriously illness	180	6	2.40 (0.94-6.10)	0.058	1.36 (0.42-4.37)	0.607
Illness and after complications	133	5	2.13 (0.78-5.80)	0.132	1.56 (0.45-5.34)	0.481
Illness, but is OK now	439	26	1.35 (0.74-2.47)	0.327	0.84 (0.41-1.72)	0.634
Nobody was ill	250	20	1.00 Ref.			
		High stress				
No, it didn't matter to me	251	29	1.00 Ref.			
No, but I am always stressed	210	10	2.43 (1.16-5.09)	0.016	4.10 (1.56-10.78)	0.004
Yes, higher	519	18	3.33 (1.82-6.11)	<0.001	2.80 (1.25-6.25)	0.012
		COVID-19 test				
Yes, many times (>3)	180	10	1.16 (0.56-2.42)	0.684	0.98 (0.34-2.84)	0.977
Yes, several times (2-3)	147	5	1.90 (0.73-4.97)	0.183	1.05 (0.32-3.44)	0.942
Yes, once	158	10	1.02 (0.49-2.12)	0.955	0.52 (0.19-1.46)	0.218

No	495	32	1.00 Ref.			
Influence of Government Campaign on vaccination decision						
Yes	68	3	2.02 (0.54-7.61)	0.288*	3.11 (0.64-15.23)	0.161
No, I was going to take the vaccine any way	459	3	13.7 (3.70-50.46)	<0.001*	12.35 (2.97-51.35)	0.001*
It didn't matter to me	112	10	1.00 Ref.			
I wasn't interested in this campaign	246	18	1.22 (0.55-2.73)	0.627	1.93 (0.72-5.13)	0.190
The information wasn't too reliable in my opinion	98	22	0.40 (0.18-0.88)	0.020	0.82 (0.29-2.26)	0.694
Where did you usually get the information about the vaccination						
Academic lectures. medical literature etc.	473	16	1.00 Ref.			
Health professionals	446	23	0.66 (0.34-1.26)	0.201	0.85 (0.37-1.96)	0.711
The Internet. the sites dedicated to COVID-19	445	20	0.75 (0.39-1.47)	0.404	2.07 (0.93-4.60)	0.075
Friends. also the social media	72	4	0.61 (0.20-1.87)	0.382	3.01 (0.72-12.66)	0.132
Radio, television, press	97	18	0.18 (0.09-0.37)	<0.001	0.16 (0.06-0.39)	<0.001
How do you rate the reliability of the different sources information on a scale of 1-5 (where 1 is unreliable. 2- not very reliable. 3- I do not known. 4- reliable. 5- very reliable – results for 1+2: not-reliable)						
Academic lectures. medical literature etc.	20	13	1.00 Ref.			
Health professionals	70	15	3.03 (1.24-7.41)	0.013	0.16 (0.06-0.39)	<0.001
The Internet. the sites dedicated to COVID-19	491	23	13.88 (6.15-31.31)	<0.001	8.37 (1.97-35.65)	0.004
Friends and the social media	734	33	14.46 (6.62-31.56)	<0.001	0.15 (0.04-0.68)	0.013
Radio, television, press	615	37	10.80 (4.99-23.41)	<0.001	0.22 (0.05-1.00)	0.049
How do you rate the reliability of the different sources information on a scale of 1-5 (where 1 is unreliable. 2- not very reliable. 3- I do not known. 4- reliable. 5- very reliable – results for 4+5: reliable)						
Academic lectures. medical literature etc.	894	32	1.00 Ref.			
Health professionals	844	33	0.92 (0.56-1.50)	0.727	0.78 (0.16-3.79)	0.759
The Internet. the sites dedicated to COVID-19	379	25	0.54 (0.32-0.93)	0.024	0.37 (0.08-1.70)	0.202
Friends. also the social media	123	14	0.31 (0.16-0.61)	<0.001	0.92 (0.22-3.83)	0.904
Radio, television, press	212	5	1.52 (0.58-3.94)	0.388	1.35 (0.43-4.26)	0.609
Should the vaccine be obligatory for the health professionals						
Obligatory	743	6	1.00 Ref.			
Voluntary	229	42	0.04 (0.02-0.10)	<0.001	0.14 (0.06-0.29)	<0.001
